# Supplementary material for: Defining the function of OmpA in the Rcs stress response
Source: eLife. 2020 Sep 28;9:e60861. doi: 10.7554/eLife.60861 (PMC7553776; doi:10.7554/eLife.60861)
Supplement: Supplementary file 1. [file elife-60861-supp1.docx]

**Supplementary Table 1.** Strains used in this study.

| Strains | Relevant Genotype or Features | Source or notes |
| --- | --- | --- |
| XL1-Blue | *endA*1 *gyrA*96(*nal*^R^) *thi*-1 *recA*1 *relA*1 *lac* *gln*V4’ F'[ ::Tn10 *proAB*^+^ *lacI*^q^ Δ(*lacZ*)M15] *hsdR*17(r_K_^-^ m_K_^+^) | Stratagene |
| BL21 (DE3) | *E. coli* str. B F^–^ *ompT* *gal* *dcm* *lon* *hsdS_B_*(*r_B_*^–^*m_B_*^–^) λ(DE3 [*lacI* *lacUV5*-*T7p07* *ind1* *sam7* *nin5*]) [*malB*^+^]_K-12_(λ^S^) | Novagen |
| SHuffle T7 | *fhuA2 lacZ::T7 gene1 [lon] ompT ahpC gal λatt::pNEB3-r1-cDsbC (SpecR, lacIq ) ΔtrxB sulA11 R(mcr-73::miniT–0--TetS )2 [dcm] R(zgb-210::Tn– --TetS ) endA1 Δgor ∆(mcrC-mrr)114::IS10* | New England Biolabs (NEB) |
| DH300 | *rprA*-*lacZ* MG1655 (*argF-lac*)*U169* | ([Majdalani, Hernandez, and Gottesman 2002](#_ENREF_38)) |
| Keio collection single mutants | Δ*rcsF*::*kan,* Δ*ompA::kan* | ([Baba et al. 2006](#_ENREF_2)) |
| PL358 | DH300 Δ*rcsF* | ([Cho et al. 2014](#_ENREF_8)) |
| SEN588 | DH300 Δ*ompA::kan* | ([Cho et al. 2014](#_ENREF_8)) |
| PR46 | DH300 Δ*ompA* | this study |
| SEN589 | PL358 Δ*ompA::kan* | ([Cho et al. 2014](#_ENREF_8)) |
| SEN524 | PL358 Δ*ompR::kan* | this study |
| SEN550 | DH300 pSC237 | this study |
| SEN860 | DH300 Δ*ompA*::*cat, sacB* | this study |
| SEN861 | PL358 Δ*ompA*::*cat, sacB* | this study |
| SEN1194 | SEN860 *ompA-6×His* | this study |
| SEN905 | SEN861 *ompA-6×His* | this study |
| SEN900 | SEN860 *ompA_TH189_-6×His* | this study |
| SEN901 | SEN860 *ompA_TH243_-6×His* | this study |
| SEN962 | SEN900 Δ*rcsF::kan* | this study |
| SEN964 | SEN901 Δ*rcsF::kan* | this study |
| SEN856 | DH300 Δ*ompA_171-325_::kan* | this study |
| SEN857 | PL358 Δ*ompA_171-325_::kan* | this study |
| SEN858 | DH300 Δ*ompA_171-325_* | this study |
| SEN859 | PL358 Δ*ompA_171-325_* | this study |
| SEN1089 | PL358 pSUP-Mb_DiZPK-RS and pSC253 | this study |
| SEN1091 | PL358 pSUP-Mb_DiZPK-RS and pSC253(Q79X) | this study |
| SEN1094 | PL358 pSUP-Mb_DiZPK-RS and pSC253(P116X) | this study |
| SEN1109 | PL358 pSUP-Mb_DiZPK-RS and pSC253(K98X) | this study |
| SEN1113 | PL358 pSUP-Mb_DiZPK-RS and pSC253(E110X) | this study |
| SEN1115 | PL358 pSUP-Mb_DiZPK-RS and pSC253(Q121X) | this study |
| SEN1129 | PL358 pSUP-Mb_DiZPK-RS and pSC253(R21X) | this study |
| SEN1130 | PL358 pSUP-Mb_DiZPK-RS and pSC253(Q28X) | this study |
| SEN1131 | PL358 pSUP-Mb_DiZPK-RS and pSC253(Q33X) | this study |
| SEN1134 | PL358 pSUP-Mb_DiZPK-RS and pSC253(R45X) | this study |
| SEN1136 | PL358 pSUP-Mb_DiZPK-RS and pSC253(N54X) | this study |
| SEN1141 | PL358 pSUP-Mb_DiZPK-RS and pSC253(R89X) | this study |
| SEN1275 | SEN589 pSUP-Mb_DiZPK-RS and pSC253(R89X) | this study |
| SEN1276 | SEN524 pSUP-Mb_DiZPK-RS and pSC253(R89X) | this study |
| SEN1689 | SEN589 pSUP-Mb_DiZPK-RS and pSC253(K98X) | this study |
| SEN1690 | SEN589 pSUP-Mb_DiZPK-RS and pSC253(E110X) | this study |
| SEN1378 | PR46 pSUP-Mb_DiZPK-RS and pPR21 | this study |
| SEN1381 | PR46 pSUP-Mb_DiZPK-RS and pPR21(R242X) | this study |
| SEN1552 | DH300 pSC231 | this study |
| SEN1562 | PR46 pSC237 | this study |
| SEN1565 | PL358 pSC237 | this study |
| PR44 | PR46 pPR4 | this study |
| JLE60 | SEN860 Δ*ompA*::*ompX-ompA_171-325_* | this study |
| JLE61 | SEN861 Δ*ompA*::*ompX-ompA_171-325_* | this study |
| KiD003 | BL21(DE3) pKiD5 | this study |
| KiD083 | PR46 pSUP-Mb_DiZPK-RS and pPR21(D246X) | this study |
| KiD084 | PR46 pSUP-Mb_DiZPK-RS and pPR21(Y248X) | this study |
| KiD123 | DH300 pKiD22 | this study |
| KiD124 | PR46 pKiD22 | this study |
