# Supplementary material for: Defining the function of OmpA in the Rcs stress response
Source: eLife. 2020 Sep 28;9:e60861. doi: 10.7554/eLife.60861 (PMC7553776; doi:10.7554/eLife.60861)
Supplement: Supplementary file 2. [file elife-60861-supp2.docx]

**Supplementary Table 2.** Primers used in this study.

| Name | Sequence (5’ to 3’) |
| --- | --- |
| OmpA(NcoI) F | gagaCCATGGgaaaaaagacagc |
| OmpA-his (XbaI) R | gagaTCTAGAtcagtggtggtggtggtggtgagcctgcggctgagttac |
| ompA_delCmSB F | atattcatggcgtattttggatgataacgaggcgcaaaaaAAAATGAGACGTTGATCGGCACG |
| ompA_delCmSB R | aaaaccccgcagcagcggggtttttctaccagacgagaacATCAAAGGGAAAACTGTCCA |
| cmSB to OmpA_F | atattcatggcgtattttggatgataacgaggcgcaaaaaATGAAAAAGACAGCTATCGCG |
| cmSB to OmpAhis_R | aaaaccccgcagcagcggggtttttctaccagacgagaacTTAGTGGTGGTGGTGGTGGTGAGCCTG |
| ompAc_delKm F | gcactcgtccggacaacggcatgctgagcctgggtgtttcctaccgtttcTAAattccggggatccgtcgacc |
| ompAc_delKm R | gcagcggggtttttctaccagacgagaacttaagcctgcggctgagttactgtaggctggagctgcttcg |
| OmpX(NcoI)F | GAGccatgggtAAAAAAATTGCATGTCTTTC |
| OmpX-OmpAc_F | GTTGGTTACCGCTTCggtcagggcgaagca |
| OmpX-OmpAc_R | gccctgaccGAAGCGGTAACCAACACCGGC |
| JLEo16-R-XbaI OmpACt | GGGtctagactaagcctgcggctgagttacaacg |
| JLEo20-F-Chrom-OmpX | atattcatggcgtattttggatgataacgaggcgcaaaaaATGAAAAAAATTGCATGTC |
| JLEo22-R-Chrom-OmpACt | aaaaccccgcagcagcggggtttttctaccagacgagaacctaagcctgcggctgagttacaacg |
| ompA -100 | cgccaggggtgctcggcataagccg |
| ompA +100 | tcttctctgaagcaggatctgcaggc |
| 189VPRGS thr_F | gctccggcaccggaagtaGTGCCGCGCGGATCCcagaccaagcacttcactc |
| 189VPRGS thr_R | gagtgaagtgcttggtctgGGATCCGCGCGGCACtacttccggtgccggagc |
| 243LVPR thr_F | ggttacaccgaccgcatcCTGGTGCCGCGCggttctgacgcttacaac |
| 243LVPR thr_R | gttgtaagcgtcagaaccGCGCGGCACCAGgatgcggtcggtgtaacc |
| KiDo14-F-NdeI-Strep-OmpACTD | GGGcatATGTGGAGCCACCCGCAGTTCGAAAAAgcaccggaagtacagacc |
| KiDo15-R-SacI-Rbs-OmpACTD | GGGGAGCTCGGCTATATCTCCTTCTTAAAGTTAAACAAAATTGCGGCCGCttaagcctgcggctgagttac |
| lacIq NsiI_F | CGAAGCGGCATGCATTTAC |
| flag3-KpnI_R | gagagggtaccCTACTTGTCATCGTCATC |
| IgaA(NcoI) F | gagaCcatggggAGCACCATTGTG |
| IgaA XbaI flag3 R | gggTCTAGAttcgataaggctttctgaagggg |
| OA_stopTGA_F | actcagccgcaggctTGAcaccaccaccaccac |
| OA_stopTGA_R | gtggtggtggtggtgTCAagcctgcggctgagt |
| Palss(NcoI)F | gagCCatgGGAcaactgaacaaagtgctg |
| Palss-OmpAc_F | GCATGTTCTTCCgaagcagc |
| Palss-OmpAc_R | ggagctgcttcGGAAGAACATGCCGCAAT |
| OmpA_stop_Flag3(KpnI)R | ggtaccCTACTTGTCATCGTCATCCTTGTAATCggatcaagcctgcggctgagttac |
| AA62_RcsF_R21x_F | ctgttccatgttaagcTAGtcccctgtcgaacc |
| AA63_RcsF_R21x_R | ggttcgacaggggaCTAgcttaacatggaacag |
| AA64_RcsF_Q28x_F | ctgtcgaacccgttTAGagcactgcaccccagc |
| AA65_RcsF_Q28x_R | gctggggtgcagtgctCTAaacgggttcgacag |
| AA66_RcsF_Q33x_F | CAAagcactgcacccTAGccgaaagcggagc |
| AA67_RcsF_Q33x_R | gctccgctttcggCTAgggtgcagtgctTTG |
| AA72_RcsF_N54x_F | cCGAatttataccTAGgcagaagaattagtcggc |
| AA73_RcsF_N54x_R | gccgactaattcttctgcCTAggtataaatTCGg |
| AA74_RcsF_R89x_F | cattccaaccgcaTAGaagcggatgcaaatcaacg |
| AA75_RcsF_R89x_R | cgttgatttgcatccgcttCTAtgcggttggaatg |
| RcsF_R45x_F | aaaccgaaagcgccgTAGgccacgccggtccga |
| RcsF_R45x_R | tcggaccggcgtggcCTAcggcgctttcggttt |
| RcsF_Q79x_F | tgccaggcctctaatTAGgactctccgccgagc |
| RcsF_Q79x_R | gctcggcggagagtcCTAattagaggcctggca |
| RcsF_K98x_F | atgcaaatcaacgcctctTAGatgaaagccaatgctgta |
| RcsF_K98x_R | tacagcattggctttcatCTAagaggcgttgatttgcat |
| RcsF_E110x_F | ttactgcatagctgcTAGgtcaccagcggtacg |
| RcsF_E110x_R | cgtaccgctggtgacCTAgcagctatgcagtaa |
| RcsF_P116x_F | gtcaccagcggtacgTAGggctgctatcgtcag |
| RcsF_P116x_R | ctgacgatagcagccCTAcgtaccgctggtgac |
| RcsF_Q121x_F | acgccaggctgctatcgtTaggctgtatgtatcggtt |
| RcsF_Q121x_R | aaccgatacatacagcctAacgatagcagcctggcgt |
| RcsF_R242x_F | ctgggttacaccgacTAGatcggttctgacgct |
| RcsF_R242x_R | agcgtcagaaccgatCTAgtcggtgtaacccag |
| KiDo87_OmpA_D246x_F | catcggttctTAGgcttacaaccagg |
| KiDo88_OmpA_D246x_R | gttgtaagcCTAagaaccgatgcgg |
| KiDo89_OmpA_Y248x_F | ttctgacgctTAGaaccagggtc |
| KiDo90_OmpA_Y248x_R | cctggttCTAagcgtcagaaccg |
