# Supplementary material for: Defining the function of OmpA in the Rcs stress response
Source: eLife. 2020 Sep 28;9:e60861. doi: 10.7554/eLife.60861 (PMC7553776; doi:10.7554/eLife.60861)
Supplement: Supplementary file 3. [file elife-60861-supp3.docx]

**Supplementary Table 3.** Plasmids used in this study.

| Plasmids | Features | Source or notes |
| --- | --- | --- |
| pDSW204 | IPTG-regulated modified P*_trc_*, ampicillin | ([Weiss et al. 1999](#_ENREF_62)) |
| pAM238 | IPTG-regulated P*_lac_*, pSC101-based, spectinomycin | ([Gil and Bouche 1991](#_ENREF_19)) |
| pBAD18 | Arabinose regulation, ampicillin | ([Guzman et al. 1995](#_ENREF_20)) |
| pSup-Mb-DIZPK-RS | PylRS*,* $\mathrm{tRNA}_{\mathrm{CUA}}^{\mathrm{Pyl}}$ _opt_, p15A origin, chloramphenicol | ([Zhang et al. 2011](#_ENREF_65)) |
| pSIM5-tet | pSC101-based, *repA^ts^*, tetracycline | ([Koskiniemi et al. 2011](#_ENREF_31)) |
| pCP20 | *FLP*^+^, λ *c*I857^+^, λ *_P_*_R_ Rep^ts^, ampicillin, chloramphenicol | ([Cherepanov and Wackernagel 1995](#_ENREF_6)) |
| pSC202 | pAM238 with RcsF | ([Cho et al. 2014](#_ENREF_8)) |
| pSC253 | pBAD18 with RcsF | this study |
| pPR11 | pDSW204 with OmpA-6×His | this study |
| pPR11_189thrombin_ | pDSW204 with OmpA(189-Val-*Val-Pro-Arg-Gly-Ser*-Gln-190)-6×His | this study |
| pPR11_243thrombin_ | pDSW204 with OmpA(243-Ile-*Leu-Val-Pro-Arg*-Gly-244)-6×His | this study |
| pJLE17-OmpX-OmpA_171-325_ | pDSW204 with OmpA_X_ | this study |
| pKiD5 | pET21a with strep-OmpA_186-325_ | this study |
| pMER77 | pDSW204 with Flag_3_ | ([Hemmis et al. 2011](#_ENREF_22)) |
| pSC231 | pAM238 with LacI^q^, the modified P*_trc_*, and Flag_3_ | this study |
| pPR4 | pSC231 with OmpA | this study |
| pSC237 | pSC231 with IgaA | this study |
| pPR21 | pDSW204 with OmpA | this study |
| pKiD22 | pDSW204 with OmpA_Pal_ | this study |
